# Established Risk Factors Account for Most of the Racial Differences in Cardiovascular Disease Mortality

**DOI:** 10.1371/journal.pone.0000377

**Published:** 2007-04-18

**Authors:** Sean O. Henderson, Christopher A. Haiman, Lynne R. Wilkens, Laurence N. Kolonel, Peggy Wan, Malcolm C. Pike

**Affiliations:** 1 Department of Preventive Medicine, Keck School of Medicine of the University of Southern California, Los Angeles, California, United States of America; 2 Department of Emergency Medicine, Keck School of Medicine of the University of Southern California, Los Angeles, California, United States of America; 3 Epidemiology Program, Cancer Research Center of Hawaii, University of Hawaii, Honolulu, Hawaii, United States of America; London School of Hygiene and Tropical Medicine, Peru

## Abstract

**Background:**

Cardiovascular disease (CVD) mortality varies across racial and ethnic groups in the U.S., and the extent that known risk factors can explain the differences has not been extensively explored.

**Methods:**

We examined the risk of dying from acute myocardial infarction (AMI) and other heart disease (OHD) among 139,406 African-American (AA), Native Hawaiian (NH), Japanese-American (JA), Latino and White men and women initially free from cardiovascular disease followed prospectively between 1993–1996 and 2003 in the Multiethnic Cohort Study (MEC). During this period, 946 deaths from AMI and 2,323 deaths from OHD were observed. Relative risks of AMI and OHD mortality were calculated accounting for established CVD risk factors: body mass index (BMI), hypertension, diabetes, smoking, alcohol consumption, amount of vigorous physical activity, educational level, diet and, for women, type and age at menopause and hormone replacement therapy (HRT) use.

**Results:**

Established CVD risk factors explained much of the observed racial and ethnic differences in risk of AMI and OHD mortality. After adjustment, NH men and women had greater risks of OHD than Whites (69% excess, P<0.001 and 62% excess, P = 0.003, respectively), and AA women had greater risks of AMI (48% excess, P = 0.01) and OHD (35% excess, P = 0.007). JA men had lower risks of AMI (51% deficit, P<0.001) and OHD (27% deficit, P = 0.001), as did JA women (AMI, 37% deficit, P = 0.03; OHD, 40% deficit, P = 0.001). Latinos had underlying lower risk of AMI death (26% deficit in men and 35% in women, P = 0.03).

**Conclusion:**

Known risk factors explain the majority of racial and ethnic differences in mortality due to AMI and OHD. The unexplained excess in NH and AA and the deficits in JA suggest the presence of unmeasured determinants for cardiovascular mortality that are distributed unequally across these populations.

## Introduction

Cardiovascular disease (CVD), which we define here to exclude stroke, continues to be one of the most common causes of mortality worldwide, and, in the U.S., it accounts for more than a quarter of all deaths. [1], [2], [3], [4] The patterns of cardiovascular mortality in individual “racial” groups have been examined and the roles of various risk factors defined. Japanese and Latino populations have been reported to have a lower than expected observed prevalence of cardiovascular disease when compared to Caucasian populations despite a similar, and in some cases increased, presence of established risk factors such as diabetes and hypertension. [5], [6], [7], [8]

Even after controlling for the established risk factors of blood pressure, serum cholesterol, cigarette smoking, and diabetes, African Americans continued to exhibit higher mortality rates from cardiovascular causes when compared to their Caucasian counterparts. [9], [10], [11], [12], [13], [14] Native Hawaiians have increased rates of obesity, a higher prevalence of diabetes, higher serum cholesterol and the highest cardiovascular mortality rate of any group in Hawaii. [15], [16], [17] The overall age- and gender-specific mortality rates due to heart disease are 66% greater in Native Hawaiians than Caucasians in the state. [18]

Using the Multiethnic Cohort (MEC), a large population-based study of adult men and women living in Hawaii and California aged 45–75 at recruitment, we have examined the mortality rates from cardiovascular causes in five racial groups, African Americans (AA), Native Hawaiians (NH), Japanese Americans (JA), Latinos (LA) and Whites (W), to specifically address whether the observed differences in CVD mortality can be explained by differences in the prevalence of established CVD risk factors.

## Methods

### Description of the Cohort

The MEC is a prospective cohort of men and women in Hawaii and California. The cohort was designed to include the five major racial groups in Hawaii and California, namely, African Americans, Native Hawaiians, Japanese Americans, Latinos and Whites. Small numbers of other ethnic groups are in the cohort because of the way the cohort was recruited.

In both locations, the MEC was assembled using primarily drivers' license files combined with master lists from the Healthcare Finance Administration. Japanese Americans and Latinos were targeted by surname using comprehensive computerized lists of Japanese and Spanish surnames from the Census Bureau supplemented by those maintained by the Hawaii and Los Angeles Surveillance Epidemiology and End Results (SEER) cancer registries. African Americans were identified by selecting census tracts in Los Angeles with a minimum proportion of individuals (85%) who identified themselves as African Americans in the 1990 census. Once contacted, individuals self assigned themselves as African American, Japanese American, Latino, Native Hawaiian or White. Persons of mixed race were assigned using the following priority ranking: African American, Native Hawaiian, Latino, Japanese American and White. It should be noted that in this cohort, >95% of every racial group, except Native Hawaiians, reported one group only [19].

The total size of the cohort is over 215,000. For the study reported here we restricted attention to the participants aged 45–75 at cohort creation in 1993 from the five main racial groups. We excluded participants who reported a history of heart attack or angina or stroke (n = 16,408) and those with missing or incomplete questionnaire information on smoking, diabetes, hypertension, physical activity, weight, height, educational level or dietary history, thus excluding a further 26,931 individuals. For females we excluded those without known information on menopausal status and use of menopausal hormone therapy (n = 5,894). Finally we excluded women who were premenopausal at recruitment (n = 13,196). This left 139,406 individuals available for study.

### Initial Questionnaire

Upon cohort enrollment, the MEC participants returned a detailed 26-page mail questionnaire that asked about diet (including alcohol intake), demographic factors, including race/ethnicity, education, longest held occupation and migrant status, personal behaviors (*e.g.*, smoking, and physical activity), anthropometry, dietary history, history of prior medical conditions (*e.g.*, hypertension, heart attack and cancer), recent medication use (*e.g.*, diuretics or specific antihypertensive drugs) and for women, reproductive history and use of exogenous hormones.

### Outcome

The cohort members are linked each year to the state death files for Hawaii and California to ascertain their vital status. Individuals residing in other states or whose current address is unknown are linked to the National Death Index file. The status of all individuals residing in California and Hawaii was ascertained up to December 31, 2003. The underlying cause of death was established from the official death certificate and was coded according to the Ninth Revision of the International Classification of Disease (ICD-9). Codes of interest were: 410 (acute myocardial infarction), 411–414 (other acute and subacute forms of ischemic heart disease, angina pectoris, other forms of chronic ischemic heart disease), and 425–429 (cardiomyopathy, unspecified cardiovascular disease, cardiomegaly, congestive heart failure, myocardial degeneration, and myocarditis). For this analysis, cause of death was divided into two categories, acute myocardial infarction (AMI: 410) and all other heart disease (OHD: 411–414, 425–429).

In addition to race and/or ethnicity, the following risk factors (as reported on the questionnaire) were evaluated in this study: body mass index (BMI, weight in kg/height in m squared), history of diabetes, history of hypertension, cigarette smoking status and pack-years, alcohol intake, amount of vigorous physical activity, educational level and diet. Vigorous activity included both vigorous sports and vigorous work. Dietary factors included saturated fat, unsaturated fat, dietary fiber from fruits, dietary fiber from grains, dietary fiber from total legumes, omega 3, omega 6, cholesterol, sodium, folate and isoflavones.

For female subjects, type of and age at menopause and history of HT use were also evaluated. Women who were continuing to menstruate naturally (or were on hormonal contraceptives) at the time of the questionnaire were excluded from this analysis. The remaining ‘postmenopausal’ women were categorized as having had a natural menopause or an oophorectomy or a simple hysterectomy. For the latter two groups of women age at ‘menopause’ was taken as the age at operation, while for a naturally postmenopausal woman the following schema was adopted. If she was not taking hormones at the time she stopped menstruating, age at menopause was taken as her age at this time. For a woman taking HT before her reported age at last menstrual period, we set the age of menopause to the age in which she began HT use (excluding use of progestins alone); with the rationale that HT use was started because of menopausal symptoms. This is the same schema to approximate age at menopause as we used in earlier studies of HRT and endometrial and breast cancer. [20], [21], [22]

### Statistical Analysis

Hazard ratios, which we report as rate ratios (RRs), for AMI and OHD mortality were estimated using log-linear proportional hazard regression models (Cox regression) as implemented in Procedure stcox in the Stata statistical software package (Stata Version 8; Stata Corporation, College Station, TX) with the risk factors categorized considered as categorical variables. The dietary risk factors described above were evaluated as nutrient densities separated into quartiles and considered as categorical variables. Tests for trend for a categorical variable were made as appropriate scoring the categorical variable as 1, 2, 3, and so on (in these cases the categorical variable was dropped from the model and replaced by the trend variable fitted as a linear term). The underlying time variable in the Cox regression was age (as a continuous variable), with observation from the date of enrollment to the date of death, or censoring (date at end of follow-up).

Mortality rates age-standardized to the US Standard Population ages 45–79 years for the year 1970 were calculated for Whites. These rates for Whites were then multiplied by the appropriate rate ratios estimated by the log-linear regression model to estimate the mortality rates in the other racial groups. These rates are given to permit the reader to have an idea of the approximate mortality rates in the different groups. Confidence intervals are given for the rate ratios as calculated by the log-linear proportional hazard regression model. Confidence intervals are not given for the mortality rates as these are only given to help the reader understand the approximate magnitude of the rates; comparisons between the racial groups are obtained from the rate ratios.

## Results

There were 572 and 1,472 deaths from AMI and OHD respectively among the 70,739 men in the five racial groups (Table 1). Compared to White males, age-adjusted death rates for AMI were highest in Native Hawaiians (RR = 1.62, P = 0.004) and in African Americans (RR = 1.54, P<0.001). The excesses for OHD mortality of Native-Hawaiian (RR = 2.09, P<0.001) and African-American (RR = 1.66 excess, P<0.001) men were still greater. In sharp contrast, mortality rates of Japanese-American men were significantly lower than the rates for Whites for both AMI and OHD (RR = 0.63, P<0.001, and RR = 0.75, P<0.001, respectively) while rates for Latino men were similar to those of Whites.

**Table 1:** Mortality rates from AMI and OHD by race and gender in the MEC.

	W	AA	NH	JA	LA	Total
**Men**						
No. of men	18,446	8,981	5,020	21,753	16,539	70,739
AMI: Deaths	143	129	49	125	126	572
Mortality rate^a^	60.2	144.4	124.5	38.5	64.4	
RR^b^	1.00	1.54	1.62	0.63	0.94	
		(1.22–1.96)	(1.17–2.25)	(0.49–0.80)	(0.74–1.20)	
		P<0.001	P = 0.004	P<0.001	P = 0.614	
OHD: Deaths	326	319	141	347	339	1,472
Mortality rate^a^	124.9	299.7	258.5	79.9	133.6	
RR^b^	1.00	1.66	2.09	0.75	1.12	
		(1.42–1.94)	(1.71–2.55)	(0.65–0.88)	(0.96–1.31)	
		P<0.001	P<0.001	P<0.001	P = 0.134	
**Women**						
No. of women	17,742	12,241	4,616	20,242	13,826	68,667
AMI: Deaths	77	146	27	68	56	374
Mortality rate^a^	21.4	51.3	44.3	13.7	22.9	
RR^b^	1.00	2.47	1.82	0.65	1.06	
		(1.87–3.25)	(1.17–2.82)	(0.47–0.90)	(0.75–1.49)	
		P<0.001	P = 0.008	P = 0.009	P = 0.752	
OHD: Deaths	168	269	72	126	125	760
Mortality rate^a^	53.8	129.1	111.3	34.4	57.5	
RR^b^	1.00	2.10	2.17	0.56	1.08	
		(1.73–2.55)	(1.65–2.87)	(0.44–0.71)	(0.85–1.36)	
		P<0.001	P<0.001	P<0.001	P = 0.532	

a Per 100,000 per year, age-standardized to US Standard Population ages 45–79 years for the year 1970. This is calculated for Ws, and the mortality rates for other groups are computed by multiplying this figure by the racial RRs.

b Rate ratios from log-linear proportional hazard regression model (Cox regression; see Statistical Analysis).

Among the 68,667 women included in this analysis there were 374 deaths due to AMI and 760 due to OHD (Table 1). As was seen in males, African Americans and Native Hawaiians had the highest mortality rates for both AMI and OHD, with more than two-fold higher mortality rates than those of Whites for OHD. All of these differences were statistically highly significant. Japanese-American women had much lower rates than Whites for both AMI (RR = 0.65, P = 0.009) and OHD (RR = 0.56, P<0.001) while rates for Latinos were similar to those of Whites.

### Distribution of Established CVD Risk Factors Among Men

The age-standardized distribution of the risk factors in men in the different racial groups is shown in Table 2. Obesity (BMI> = 30.0) was least common in Japanese Americans (7.8%), but was 15.5% in Whites, 19.5% in Latinos, 21.4% in African Americans and 31.8% in Native Hawaiians. Hypertension was reported by between a third and slightly more than a half of men in the different ethnic groups from the lowest proportion in Whites (31.0%), through Latinos (33.2%), Japanese Americans (42.1%) and Native Hawaiians (48.9%), to the highest proportion in African Americans (51.9%). Similar large differences were seen for diabetes; the disease was most frequently reported by African Americans (13.3%), Native Hawaiians (15.7%) and Latinos (14.2%), at more than 2.5 times the frequency in Whites (5.4%), with Japanese Americans at an intermediate frequency (10.4%). Current smoking was more frequent in the African Americans but the Native Hawaiians had more current smokers with a greater than 20 pack-years history. High levels of alcohol intake (>24 g/day) were seen most commonly in Whites (27.8%), approximately 50–60% more frequently than in the other racial groups. Vigorous physical activity was reported least often in the African Americans (65.0%) and most often in the Native Hawaiians (77.6%).

**Table 2:** Age-standardized^a^ distributions of risk factors with associated RRs of mortality from AMI and OHD among men in the MEC.

	W	AA	NH	JA	LA	AMI		OHD	
Men	%	%	%	%	%	RR^b^	P	RR^b^	P
BMI (kg/m^2^)									
<23	17.4	13.5	10	23.9	10.2	1.35 (1.04–1.77)		1.18 (1.00–1.39)	
23−	21.6	16.8	12.8	26.1	16.4	1.00		1.00	
25−	45.5	48.3	45.4	42.2	53.8	0.92 (0.73–1.16)		0.91 (0.78–1.04)	
30−	12.2	16.5	21.4	6.7	15.6	1.02 (0.75–1.37)		1.01 (0.84–1.22)	
> = 35	3.3	4.9	10.4	1.1	3.9	1.42 (0.95–2.11)	P_t_ = 0.932	1.39 (1.08–1.80)	P_t_ = 0.768
Hypertension^c^	31.0	51.9	48.9	42.1	33.2	2.29 (1.91–2.74)	P<0.001	1.73 (1.55–1.93)	P<0.001
Diabetes^c^	5.4	13.3	15.7	10.4	14.2	2.17 (1.79–2.63)	P<0.001	2.20 (1.95–2.49)	P<0.001
Smoking									
Never	33.6	26.8	32.3	30.4	34.2	1.00		1.00	
Past< = 20 packyrs	31.4	34.9	30.9	36.3	39.8	0.98 (0.79–1.22)	P = 0.862	1.04 (0.90–1.20)	P = 0.554
Past>20 packyrs	19.0	10.5	16.2	17.3	7.5	1.16 (0.90–1.50)	P = 0.244	1.45 (1.24–1.71)	P<0.001
Current< = 20 packyrs	4.8	16.5	7.5	5.9	12.5	1.65 (1.21–2.25)	P = 0.002	1.89 (1.56–2.30)	P<0.001
Current>20 packyrs	11.1	11.3	13.1	10.0	5.9	2.11 (1.60–2.78)	P<0.001	2.79 (2.36–3.30)	P<0.001

	W	AA	NH	JA	LA	AMI		OHD	
Men	%	%	%	%	%	RR	P	RR	P
Alcohol (g eth/d)									
0	27.0	40.9	40.5	42.7	33.9	1.00		1.00	
>0–12	31.6	32.3	28.5	29.7	38.5	0.81 (0.66–0.98)		0.77 (0.68–0.87)	
>12–24	13.5	9.8	10.4	10.7	11.0	0.76 (0.56–1.02)		0.61 (0.49–0.75)	
>24	27.8	17.0	20.5	16.9	16.6	0.61 (0.47–0.79)	P_t_<0.001	0.86 (0.74–1.00)	P_t_ = 0.002
Vigorous Physical Activity (hrs/wk)									
0	27.1	35.0	22.4	32.8	30.3	1.00		1.00	
>0–1.5	14.9	16.6	13.9	18.9	15.1	0.80 (0.63–1.02)		0.82 (0.71–0.95)	
>1.5–5.0	24.6	22.9	26.3	24.4	21.9	0.79 (0.63–0.99)		0.79 (0.69–0.91)	
>5.0	33.3	25.5	37.3	23.9	32.6	0.61 (0.47–0.79)	P_t_<0.001	0.72 (0.62–0.83)	P_t_<0.001
Education									
≤High school	21.4	37.3	53.1	33.3	62.4	1.00		1.00	
Some further educ	28.8	37.9	28.9	31.1	24.5	0.87 (0.71–1.06)		0.97 (0.86–1.09)	
College graduate	49.8	24.8	18	35.5	13.1	0.75 (0.59–0.94)	P_t_ = 0.010	0.76 (0.65–0.88)	P_t_ = 0.001
Percent of calories from saturated fat									
< = 7.12	20.5	14.3	27.3	41.8	12.9	1.00		1.00	
< = 8.92	23.7	19.9	28.5	31.1	20.2	1.28 (0.98–1.66)		1.06 (0.90–1.24)	
< = 10.74	26.5	26.8	26.4	19.1	29.6	1.40 (1.07–1.81)		1.25 (1.07–1.46)	
>10.74	29.3	39.0	17.9	8.1	37.3	1.60 (1.23–2.08)	P_t_ = 0.001	1.50 (1.28–1.76)	P_t_<0.001

a Age standardized (5-year age groups) to the total male population included in the study.

b RRs for all risk factors shown estimated simultaneously (i.e. adjusted for each other).

c RRs compared to “No Hypertension”, or “No Diabetes”.

Abbreviations: eth = ethyl alcohol; P_t_ = P for trend.

There were increases in risk for both AMI and OHD mortality in men with low BMI (<23 kg/m^2^) and in those with high BMI (> = 35 kg/m^2^) (Table 2). Hypertension, diabetes and smoking were associated with significant increases in both AMI and OHD mortality. Increasing alcohol intake was associated with a steady and significant decrease in AMI mortality. Alcohol intake was clearly associated with decreased OHD mortality, but the evidence for a dose-response was unclear. Vigorous activity was associated with significant decreases in mortality from both causes. A significant inverse association was observed with level of education, with college graduates having 25% and 24% lower rates of AMI and OHD mortality, respectively.

The effects of saturated fat were as expected with higher consumption associated with higher mortality from disease in all the racial groups. The percentages of calories from saturated fat were highest in the White, African-American and Latino populations and dramatically lower in the Japanese-Americans.

### Distribution of Established CVD Risk Factors Among Women

Table 3 shows the age-standardized patterns of risk factors by racial group for women. Obesity (BMI> = 30 kg/m^2^) was least common in Japanese-American women (6.3%); this compared to prevalences from 17.0% in Whites to 35.3% in African Americans. Hypertension was as common as in men. Whites, Japanese Americans and Latinas reported the lowest proportions of hypertensives (34.6%, 37.9% and 39.1%, respectively), with very high rates in Native Hawaiians (50.0%) and African Americans (59.4%). Diabetes also showed the same pattern as in males, with the lowest proportion reported by Whites (4.9%) and the highest by African Americans, Native Hawaiians and Latinas (∼13.5%). The pattern of smoking by ethnic group in women was vastly different from that in men. Approximately two-thirds of Japanese-American and Latino women had never smoked compared to ∼45% of the other ethnic groups. African American smokers had smoked slightly less than White smokers. Alcohol use was very uncommon in Japanese-American females (22.8%), while ∼40% of African Americans, Native Hawaiians and Latinas, and 60.8% of Whites reported drinking, with the White women drinking more than the other groups.

**Table 3:** Age-standardized^a^ distributions of risk factors with associated RRs of mortality from AMI and OHD among women in the MEC.

	W	AA	NH	JA	LA	AMI		OHD	
Women	%	%	%	%	%	RR^b^	P	RR^b^	P
BMI (kg/m^2^)									
<23	35.0	13.3	19.7	49.0	16	1.01 (0.71–1.44)		1.17 (0.94–1.47)	
23−<25	19.5	13.9	16.1	21.0	17.8	1.00		1.00	
25−<30	28.6	37.5	33.9	23.7	39.5	1.05 (0.76–1.45)		0.78 (0.63–0.98)	
30−<35	11.2	21.9	18.3	5.1	18.4	1.15 (0.79–1.67)		0.79 (0.61–1.03)	
> = 35	5.8	13.4	12.0	1.2	8.3	1.45 (0.96–2.18)	P_t_ = 0.093	1.04 (0.78–1.38)	P_t_ = 0.167
Hypertension^c^	34.6	59.4	50.0	37.9	39.1	2.55 (1.98–3.27)	P<0.001	2.17 (1.84–2.56)	P<0.001
Diabetes^c^	4.9	13.1	13.6	8.5	13.7	3.25 (2.59–4.09)	P<0.001	3.34 (2.84–3.93)	P<0.001
Smoking									
Never	44.9	45.9	48.1	68.4	66.7	1.00		1.00	
Past< = 20 packyrs	27.8	28.2	24.2	19.1	21.2	1.06 (0.81–1.39)	P = 0.673	1.20 (0.99–1.45)	P = 0.068
Past>20 packyrs	10.7	5.3	8.1	3.3	1.7	1.24 (0.79–1.93)	P = 0.350	1.99 (1.52–2.60)	P<0.001
Current< = 20 packyrs	6.0	14.6	11	5.5	8.1	2.44 (1.75–3.40)	P<0.001	2.71 (2.16–3.40)	P<0.001
Current>20 packyrs	10.6	6.0	8.7	3.7	2.2	3.00 (2.09–4.33)	P<0.001	3.18 (2.49–4.07)	P<0.001

	W	AA	NH	JA	LA	AMI		OHD	
Women	%	%	%	%	%	RR	P	RR	P
Alcohol (g eth/d)									
0	39.2	60.9	64.5	77.2	63.8	1.00		1.00	
>0–12	37.9	29.8	26.2	19.4	31.0	0.83 (0.64–1.09)		0.77 (0.64–0.93)	
>12	22.9	9.3	9.3	3.4	5.3	0.67 (0.44–1.03)	P_t_ = 0.038	0.99 (0.77–1.27)	P_t_ = 0.212
Vigorous Physical Activity (hrs/wk)									
0	52.7	63.3	48.7	63.5	61.6	1.00		1.00	
>0–1.5	16.3	17.6	18	16.1	16.2	0.72 (0.52–1.00)		0.81 (0.66–1.01)	
>1.5–5.0	16.9	11.9	19.1	13.2	13	0.82 (0.56–1.19)		0.71 (0.54–0.92)	
>5.0	14.2	7.2	14.2	7.3	9.1	0.88 (0.56–1.36)	P_t_ = 0.156	0.49 (0.34–0.72)	P_t_<0.001
Education									
≤High School	30.8	37.2	59.2	40.6	70.9	1.00		1.00	
Some further educ	33.8	38.2	26.6	30.5	20.2	0.80 (0.62–1.03)		0.92 (0.77–1.10)	
College graduate	35.4	24.6	14.2	28.9	8.9	0.72 (0.53–0.97)	P_t_ = 0.017	1.05 (0.86–1.29)	P_t_ = 0.832
Menopausal Status									
Nat< = 44	8.8	9.8	11.6	7.5	13.6	1.00		1.00	
Nat 45–49	21.3	16.6	19.8	19.5	22.2	1.02 (0.71–1.47)		0.88 (0.69–1.14)	
Nat 50–54	26.2	19.5	23.8	32.9	23.6	0.88 (0.61–1.26)		0.76 (0.60–0.98)	
Nat> = 55	6.3	5.7	8.0	8.4	5.3	0.65 (0.39–1.07)		0.79 (0.57–1.09)	
Ooph< = 44	10.1	13	10.3	8.6	8.0	0.76 (0.48–1.21)		0.84 (0.62–1.14)	
Ooph 45–49	5.2	5.2	5.5	6.4	3.9	0.71 (0.38–1.34)		0.87 (0.58–1.29)	
Ooph> = 50	2.8	2.3	3.3	3.1	2.2	1.03 (0.55–1.93)		0.56 (0.33–0.98)	
Hyst< = 44	14.3	22.2	12.5	9.3	15.9	0.73 (0.48–1.10)		0.68 (0.51–0.90)	
Hyst 45–49	3.5	4.2	3.6	2.8	3.6	0.85 (0.47–1.54)		0.88 (0.59–1.31)	
Hyst> = 50	1.4	1.4	1.7	1.5	1.7	1.14 (0.57–2.27)		0.81 (0.47–1.41)	
HT use^c^									
Never	34.3	54.1	50.0	39.1	52.9	1.00		1.00	
Past ET	17.7	20.7	17	13.2	17.2	1.00 (0.85–1.19)	P = 0.984	1.00 (0.89–1.13)	P = 0.975
Past PT	4.0	2.5	3.2	2.8	3.3	1.30 (0.55–3.05)	P = 0.545	0.57 (0.20–1.62)	P = 0.289
Past EPT	11.8	6.4	7.9	8.6	7.5	0.99 (0.66–1.49)	P = 0.974	0.97 (0.73–1.28)	P = 0.815
Current ET	25.1	16.2	17.6	22.3	16.8	0.90 (0.80–1.01)	P = 0.080	0.89 (0.82–0.97)	P = 0.006
Current PT	0.6	0.4	0.5	0.8	0.6	0.27 (0.01–8.86)	P = 0.459	0.61 (0.19–1.98)	P = 0.405
Current EPT	19.5	5.7	12.0	22.0	9.1	0.97 (0.78–1.21)	P = 0.807	0.66 (0.52–0.84)	P = 0.001
Percent of calories from saturated fat									
< = 6.74	21.3	16.2	24.0	40.0	14.0	1.00		1.00	
< = 8.49	23.8	21.7	27.7	31.0	20.6	0.83 (0.61–1.12)		1.16 (0.92–1.47)	
< = 10.32	25.7	26.9	27.8	20.5	28.7	0.97 (0.72–1.30)		1.48 (1.18–1.85)	
>10.32	29.2	35.1	20.6	8.2	36.7	0.84 (0.62–1.15)	P_t_ = 0.484	1.42 (1.13–1.79)	P_t_ = 0.001

a Age standardized (5-year age groups) to the total female population included in the study.

b RRs for all risk factors shown estimated simultaneously (i.e. adjusted for each other).

c RRs compared to “No Hypertension”, or “No Diabetes”.

d Per 5 years of use.

Abbreviations: eth = ethyl alcohol; Nat = natural menopause; Ooph = oophorectomy; P_t_ = P for trend.

Hypertension, diabetes and current smoking increased CVD mortality risk (Table 3), as expected, and this was true in all racial groups (data not shown). Obesity appeared to have no significant effect on mortality except for AMI in those with BMI> = 35 kg/m^2^. The associations of AMI and OHD with alcohol use were not straightforward; although the results point to some degree of reduced risk in drinkers compared to non-drinkers, there was no evidence of a dose-response effect. Vigorous activity was associated with a lower mortality from both AMI and OHD, but only OHD showed a dose-response relationship. Unlike among men, among women level of attained education was only associated with a significantly lower AMI mortality. Although the distribution of the percentage of calories from saturated fat was similar to men in that Whites, African Americans and Latinos had the highest percentage, unlike men, there was no association between increasing percentage of dietary calories from saturated fat and mortality from AMI. The association with OHD mortality remained.

Risk of death from both AMI and OHD showed fairly consistent steady declines with increasing age at natural menopause (Table 3). There was no particular relationship between age at oophorectomy or simple hysterectomy and either outcome. Current ET and EPT use were associated with a significant decrease in OHD mortality but not in AMI mortality.

### Race

Table 4 shows the rate ratios of CVD mortality from AMI and OHD for men and women for the different racial groups relative to Whites ‘adjusted’ for age and the non-dietary risk factors shown in Tables 2 and 3, and additionally for the 11 dietary factors listed in the Research Design and Methods section above.

**Table 4:** Observed and adjusted rate ratios (RRs) of death from AMI and OHD by race and gender in the MEC.

	W	AA	NH	JA	LA
**Men**					
**AMI**					
RR	1.00	1.54	1.62	0.63	0.94
		(1.22–1.96)	(1.17–2.25)	(0.49–0.80)	(0.74–1.20)
		P<0.001	P = 0.004	P<0.001	P = 0.614
RR adjusted for risk factors	1.00	0.98	1.08	0.45	0.74
		(0.76–1.26)	(0.77–1.52)	(0.35–0.58)	(0.57–0.96)
		P = 0.884	P = 0.651	P<0.001	P = 0.022
RR adjusted for risk factors & diet	1.00	0.94	1.17	0.49	0.74
		(0.73–1.22)	(0.81–1.67)	(0.36–0.67)	(0.56–0.97)
		P = 0.661	P = 0.403	P<0.001	P = 0.031
**OHD**					
RR	1.00	1.66	2.09	0.75	1.12
		(1.42–1.94)	(1.71–2.55)	(0.65–0.88)	(0.96–1.31)
		P<0.001	P<0.001	P<0.001	P = 0.134
RR adjusted for risk factors	1.00	1.16	1.50	0.60	0.96
		(0.99–1.37)	(1.22–1.84)	(0.51–0.70)	(0.81–1.13)
		P = 0.068	P<0.001	P<0.001	P = 0.616
RR adjusted for risk factors & diet	1.00	1.15	1.69	0.73	0.90
		(0.97–1.35)	(1.36–2.11)	(0.61–0.89)	(0.76–1.08)
		P = 0.105	P<0.001	P = 0.001	P = 0.264
**Women**					
**AMI**					
RR	1.00	2.47	1.82	0.65	1.06
		(1.87–3.25)	(1.17–2.82)	(0.47–0.90)	(0.75–1.49)
		P<0.001	P = 0.008	P = 0.009	P = 0.752
RR adjusted for risk factors	1.00	1.45	1.13	0.57	0.72
		(1.07–1.95)	(0.72–1.77)	(0.40–0.81)	(0.50–1.04)
		P = 0.015	P = 0.600	P = 0.002	P = 0.081
RR adjusted for risk factors & diet	1.00	1.48	1.24	0.63	0.65
		(1.09–2.01)	(0.77–2.01)	(0.41–0.96)	(0.44–0.96)
		P = 0.013	P = 0.380	P = 0.034	P = 0.032
**OHD**					
RR	1.00	2.10	2.17	0.56	1.08
		(1.73–2.55)	(1.65–2.87)	(0.44–0.71)	(0.85–1.36)
		P<0.001	P<0.001	P<0.001	P = 0.532
RR adjusted for risk factors	1.00	1.41	1.60	0.53	0.93
		(1.14–1.73)	(1.20–2.13)	(0.41–0.68)	(0.72–1.20)
		P = 0.001	P = 0.001	P<0.001	P = 0.584
RR adjusted for risk factors & diet	1.00	1.35	1.62	0.60	0.88
		(1.09–1.67)	(1.18–2.21)	(0.44–0.81)	(0.67–1.15)
		P = 0.007	P = 0.003	P = 0.001	P = 0.359

#### Men and AMI:

The 54% excess mortality rate in African Americans and 62% excess in Native Hawaiians largely disappeared after accounting for the risk factors to a 6% deficit for African Americans and a 17% excess for Native Hawaiians. The rates in Japanese Americans and Latinos were reduced further by adjustment for the risk factors - from 37% to 51% for Japanese Americans (P<0.001); and from 6% to 26% for Latinos (P = 0.031), when compared to Whites.

#### Men and OHD:

Following adjustment for the risk factors, the 109% excess mortality rate in Native Hawaiians was reduced by roughly one third to a 69% excess compared to Whites (P<0.001) while the 66% excess rate in African Americans was reduced to a non-significant 15% excess (P = 0.105). The mortality rate for Latinos remaining non-significantly different from Whites, while the 25% reduced rate in Japanese Americans remained significantly lower than Whites (P = 0.001) following adjustment for the risk factors.

#### Women and AMI:

The 147% excess mortality rate in African Americans was reduced to a 48% excess (P = 0.013) after adjusting for the risk factors. The 82% excess rate in Native Hawaiians was similarly reduced to a 24% excess, which was not statistically significant (P = 0.380). Rates for Japanese Americans and Latinos were similar and approximately 35% lower than in Whites (P∼0.03).

#### Women and OHD:

The large and highly significant 110% and 117% excess mortality rates in African Americans and Native Hawaiians were reduced to 35% (P = 0.007) and 62% (P = 0.003) excess rates, respectively, after adjusting for the risk factors. The rate in Latinas remained close to that of Whites after adjustment while Japanese-American women continued to have significantly lower rates than White women (40%, P = 0.001).

## Discussion

In this study we found considerable variation in mortality rates for AMI and OHD by racial group among both men and women. Similar to previous reports, the mortality rates for both AMI and OHD were highest in Native Hawaiians and African Americans and lowest in Japanese Americans.

The excess cardiovascular mortality in African Americans is well established. [9], [10], [11], [12], [13] Even after controlling for established risk factors of blood pressure, serum cholesterol, cigarette smoking, and diabetes, African Americans continued to exhibit higher mortality rates from cardiovascular causes when compared to their Caucasian counterparts. [12], [13], [14] In this cohort the African Americans' excess rates persisted in women but not in men.

In the few studies comparing Native Hawaiians to non-Hawaiians, Native Hawaiians have exhibited up to a 44% higher mortality rate from cardiovascular disease. [16], [23] Diabetes, obesity, the Insulin Resistance Syndrome, and hypertension are the risk factors most commonly cited as contributing to their excess cardiovascular deaths. [21], [22]

Finally, the Japanese have consistently demonstrated lower rates of cardiovascular mortality when compared to White populations. [6], [8], [24], [25] These findings persist despite higher rates of hypertension and smoking prevalence among Japanese populations. [8]

In this cohort, diabetes and hypertension were the most important risk factors for the increased AMI and OHD mortality in Native-Hawaiian and African-American men with smoking also contributing to their high rates. These factors, in fact, suffice to ‘explain’ the high rate of AMI mortality in African-American men and explained most of their excess of OHD. These factors also explained a significant proportion of the excess mortality from AMI and OHD in Native-Hawaiian men, but an excess mortality rate, for OHD in particular (69% excess), still remained when compared to Whites. Diabetes and hypertension were also the most important risk factors for AMI and OHD mortality in Native Hawaiian and African-American women with lower use of HRT also contributing to their increased rates but to a much lesser extent (see below). These factors explained a significant proportion of the excess mortality from AMI and OHD in Native-Hawaiian and African-American women, but substantial excesses remained for both AMI and OHD.

In contrast, the decreased mortality rates in Japanese men and women were not explained at all by the risk factors we studied, while Latinos, who have a lower prevalence of risk factors, had rates that were modestly lower than Whites in the multivariate adjusted model.

There was a substantial reduction in OHD mortality in women among current users of ET and of EPT in all racial groups (data not shown) and no reduction in AMI mortality. In the Women's Health Initiative (WHI) clinical trial of ET, there was a reduction in risk of CVD incidence in agreement with the results we observed, but in the WHI trial of EPT, there was an increased risk of AMI in apparent contradiction both to the results we observed and to the beneficial effects seen in observational case-control and cohort studies. [26], [27] In the WHI EPT trial, there was a reduction in AMI in those women who started taking EPT in the trial within 10 years of menopause; it was only in the women who started EPT 10 years or more after menopause that an increased risk was seen. [26] Further RCT are needed to firmly establish whether this “time from menopause” effect is real.

In men and women in this cohort, the protective effect of alcohol consumption was seen in all racial groups (data not shown), findings consistent with those reported by others. [28], [29], [30] In the MEC, the protective effect of alcohol was not dependent on the type of alcohol (red wine, beer, grain alcohol) consumed. While our results demonstrate an essentially linear decrease in AMI and OHD mortality with increasing alcohol consumption, there have been other reports indicating that this decreasing trend eventually begins to reverse. [28], [30] It is possible that alcohol consumption was too infrequent in the MEC to study this phenomenon. Alcohol was also associated with a significant and consistent decrease in mortality from more chronic forms of heart disease (OHD) in all the racialities studied.

The relationship between physical activity and the risk of CVD has been well described in the past. [31], [32], [33], [34], [35] As was seen in this cohort, there appears to be an inverse relationship between physical activity and risk of cardiovascular death, a relationship that is linear to a point after which the risk may increase. [33], [35] The number of men in the “>5 hour/week” for vigorous activity is higher than previously described as we combined vigorous work with sporting activities. Women demonstrate a more typical distribution. We examined vigorous physical activity specifically as such activity has been associated more often with measurable benefit as opposed to so-called non-conditioning activities such as walking.

Elevated lipid levels is a marker of CVD risk and previous studies have shown Japanese Americans to have a more favorable lipid profile than Native Hawaiians which might contribute to their decreased cardiovascular mortality. [8], [36] In contrast, multiple studies in African Americans have not demonstrated lipid profiles to be different than those of Whites, and therefore does not explain their increased cardiovascular mortality. [37], [38], [39] While we were unable to assess individual lipid levels in the present study, when the data was adjusted for intake of dietary saturated fat (as a percentage of their total caloric intake), there were only minor changes in AMI and OHD relative mortality rates.

In previous studies, socioeconomic status (SES) as measured by median income has been shown to partially explain differences in CVD mortality between African Americans and Whites, with poor living conditions and limited access to health care experienced by those in lower socioeconomic strata hypothesized to be responsible for the majority of the excess mortality between these populations. [9], [40] Our study used attained education as a proxy for SES and found that having a higher educational level was associated with significantly reduced risks of AMI and OHD in men, and with a slightly reduced risk of AMI in women. The adjusted results shown in Table 4 include adjustment for educational level, which varies considerably between the racial groups. We also classified the cohort residing in Los Angeles County (mainly African Americans and Latinos) by SES based on their census tract of residence at the time of initial questionnaire to confirm that educational level was a suitable proxy for SES and the two were highly correlated across both groups. However, neither educational level nor SES as measured by census tract of residence was associated with the excess risk of death due to AMI or OHD in African Americans.

One apparent limitation of this study is the possibility that the use of a recall of a diagnosis of hypertension in the cohort will result in an underestimation of the extent that blood pressure differences can account for mortality differences. In fact, when compared to other published data [17], [41] the prevalence of hypertension in the MEC is much higher for Whites, African Americans, Native Hawaiians and Latinos than previously reported. Therefore we do not believe we are underestimating the prevalence nor the differences between racial and racial groups as both are higher than reported elsewhere.

In summary, we found that traditional risk factors explain the majority of the differences in mortality due to AMI and OHD among these five groups. After adjustment there remained significant excess in mortality due to AMI and OHD in African-American women, and OHD in Native-Hawaiian men and women. There was also a lower than expected mortality from AMI and OHD in Japanese-American men and women, and of AMI in Latino men and women compared to Whites. For these groups there appear to be unmeasured environmental factors, social or cultural factors, or genetic risk factors for cardiovascular mortality that are distributed unequally across these populations.
